# Spatial transcriptomics atlas reveals the crosstalk between cancer-associated fibroblasts and tumor microenvironment components in colorectal cancer

**DOI:** 10.1186/s12967-022-03510-8

**Published:** 2022-07-06

**Authors:** Zhiwei Peng, Manping Ye, Huiming Ding, Zhenyou Feng, Kongwang Hu

**Affiliations:** 1 Department of General Surgery, The First Affiliated Hospital of Anhui Medical University, Hefei, 230022 Anhui China; 2 Anhui Province Key Laboratory of Major Autoimmune Diseases, Anhui Institute of Innovative Drugs, School of Pharmacy, Anhui Medical University, Hefei, 230032 Anhui China

**Keywords:** Spatial transcriptomics, Colorectal cancer, Cancer-associated fibroblasts, Tumor microenvironment

## Abstract

**Background:**

The tumor-promoting role of tumor microenvironment (TME) in colorectal cancer has been widely investigated in cancer biology. Cancer-associated fibroblasts (CAFs), as the main stromal component in TME, play an important role in promoting tumor progression and metastasis. Hence, we explored the crosstalk between CAFs and microenvironment in the pathogenesis of colorectal cancer in order to provide basis for precision therapy.

**Methods:**

We integrated spatial transcriptomics (ST) and bulk-RNA sequencing datasets to explore the functions of CAFs in the microenvironment of CRC. In detail, single sample gene set enrichment analysis (ssGSEA), gene set variation analysis (GSVA), pseudotime analysis and cell proportion analysis were utilized to identify the cell types and functions of each cell cluster. Immunofluorescence and immunohistochemistry were applied to confirm the results based on bioinformatics analysis.

**Results:**

We profiled the tumor heterogeneity landscape and identified two distinct types of CAFs, which myo-cancer-associated fibroblasts (mCAFs) is associated with myofibroblast-like cells and inflammatory-cancer-associated fibroblasts (iCAFs) is related to immune inflammation. When we carried out functional analysis of two types of CAFs, we uncovered an extensive crosstalk between iCAFs and stromal components in TME to promote tumor progression and metastasis. Noticeable, some anti-tumor immune cells such as NK cells, monocytes were significantly reduced in iCAFs-enriched cluster. Then, ssGSEA analysis results showed that iCAFs were related to EMT, lipid metabolism and bile acid metabolism etc. Besides, when we explored the relationship of chemotherapy and microenvironment, we detected that iCAFs influenced immunosuppressive cells and lipid metabolism reprogramming in patient who underwent chemotherapy. Additionally, we identified the clinical role of iCAFs through a public database and confirmed it were related to poor prognosis.

**Conclusions:**

In summary, we identified two types of CAFs using integrated data and explored their functional significance in TME. This in-depth understanding of CAFs in microenvironment may help us to elucidate its cancer-promoting functions and offer hints for therapeutic studies.

**Supplementary Information:**

The online version contains supplementary material available at 10.1186/s12967-022-03510-8.

## Introduction

As one of the most common tumors in the world, it is estimated that more than 1.9 million new colorectal cancer (CRC) cases and more than 935,000 deaths in 2020 by the International Agency for Research on Cancer (IARC), ranking third in incidence and second in mortality among known cancers [1]. Though substantial progress has been made in cancer pathogenesis and drug therapy, the clinical prognosis of colorectal cancer is still poor due to tumor metastasis [2].

In recent years, the tumor microenvironment (TME) has become a research hotspot of mechanisms of tumor biology and drug development. The tumor microenvironment is a complex system that contains a variety of cellular and noncellular components, such as: immune cells, endothelial cells, cancer-associated fibroblasts (CAFs) and cytokines et al. [3, 4]. More and more evidences indicate that the crosstalk between various stromal components in TME and tumor cells are critical factors affecting tumor growth and metastasis [5]. CAFs, as one of the most abundant stromal cell types in TME, can remodel the extracellular matrix (ECM), and promote tumor progression through the interactions with tumor cells and immune cells by secreting various growth factors, chemokines and cytokines [3]. Some studies have identified different types of CAFs at the single-cell level according to unique gene signatures or functions. However, the types and functions of CAFs varies in different studies or tumor types, suggesting that the functional roles of CAFs in TME is complicated and yet has not been clearly explained, so it is worth for further exploration [6–8].

In this research, we analyzed previously published spatial transcriptomics (ST) data and profiled a spatial atlas of TME inside colorectal cancer tissues [9]. Our work highlights the role of CAFs in colorectal cancer. In detail, we combined spatial transcriptomics with ssGSEA to precisely identify different cell types. We found that there were subsets enriched with inflammatory-cancer-associated fibroblasts (iCAFs) and myo-cancer-associated fibroblasts (mCAFs) in CRC with bioinformatics analysis and experimental verification. Through functional enrichment analysis, the functional role of CAFs in TME were further investigated. In addition, we explored the correlation between CAFs and prognosis by analyzing bulk RNA-sequencing in public dataset. These results promote an in-depth understanding of the functions of CAFs in TME and provide a basis for CRC precision therapy.

## Methods

### Data sources

The spatial transcriptomics dataset (10X Genomics) was downloaded from a spatial transcriptomics research website (http://www.cancerdiversity.asia/scCRLM/). The spatial transcriptomics data of two patients were used, one of whom did not receive neoadjuvant chemotherapy treatment (NACT) was named colon1 (ST-P1), and the other received neoadjuvant chemotherapy with partial response (PR) was named colon2 (ST-P3) [9]. Bulk RNA-sequencing dataset was derived from the COADREAD cohort of The Cancer Genome Atlas (TCGA) and downloaded from UCSC XENA (https://xena.ucsc.edu/).

### Spatial transcriptomics data processing

We used the R package Seurat (v4.1.0) to process space transcriptomics data and used log-normalization to standardize data [10]. We used functions SelectIntegrationFeatures, FindIntegrationAnchors, and IntegrateData to remove batch effects and integrate Seurat object into a single ST dataset. To reduce the dimensionality of ST data, function RunPCA was performed, then functions FindNeighbors and FindClusters were used to cluster similar ST spots. Different clusters were preliminarily annotated based on hematoxylin–eosin staining (H&E) sections and unsupervised clustering analysis. When we used cell markers to annotate clusters, we found that some clusters highly expressed multiple cell markers, so ssGSEA algorithm [11] were performed on scoring common cell types based on the average expression matrix of different clusters, and studies have confirmed its robustness in ST [12, 13].

### Enrichment analysis

Differentially expressed genes (DEGs) of cell clusters were identified by the function FindAllMarkers, and top 10 DEGs of each cluster that were ranked according to the log2FC were applied to data visualization. Function clusterProfiler (v3.18.1) [14] was used for KEGG pathway analysis. ssGSEA (Hallmark Gene sets from Molecular Signatures Database, MSigDB) was conducted with GSVA (v1.38.2) [15]. We set adj.*p*.val < 0.05, |avg. logFC|> 0.5 as cut-off criteria.

### Bulk RNA-seq data analysis

We downloaded TCGA colorectal cancer cohort data (COADREAD) for transcriptome analysis. We selected the top 10 DEGs in the cluster as cluster-specific geneset, and calculated the scores of genesets in the transcriptomic data through GSVA, median was set as cut-off value. R packages survival (v3.2-10), survminer (0.4.9) were used for survival analysis. In addition, ImmuneScore and StromalScore were calculated using ESTIMATE algorithm, and their correlation with specific cell type was analyzed by correlation analysis, *p* value < 0.05 as statistically significant.

### Pseudotime analysis

In order to analyze the cell type differentiations in the tumor microenvironment, monocle2 (v2.18.0) [16] was used for trajectory analysis to find the transitional relationships among different clusters. The plot_genes_in_pseudotime function was applied to discover transitional changes in gene expression levels among different clusters.

### Immunofluorescence assay

Specific proteins expression of distinct cancer-associated fibroblasts within colorectal cancer were analyzed using Immunofluorescence staining. Paraffin-embedded colorectal cancer tissues (n = 2) were made slices, heat mediated antigen retrieval with TRIS–EDTA (pH = 8) for 20 min and then blocked with 5% BSA for 30 min at room temperature. After incubated with anti-PDGFRA (rabbit, 1:100, HuaBio, ET1702-49) and anti-RGS5 (rabbit, 1:300, Affinity, Cat. #: DF4417) separately at 4℃ overnight, the sections were washed with PBS for three times, then 488 conjugated goat anti-rabbit IgG (1:800 dilution, HA1121) and 594 conjugated goat anti-rabbit IgG (1:800 dilution, HA1122) were separately added and incubated for 1 h. Finally, DAPI was applied to stain cell nucleus. Sections without incubation with primary antibody or secondary antibody were used as control (without any light).

### Immunohistochemical analysis

Immunohistochemical analysis was carried out to explore the specific protein expression in distinct tissues. Paraffin-embedded colorectal cancer or para-carcinoma tissues (n = 5) were applied in this project. Sections were firstly treated with boiling TRIS–EDTA (pH = 8) to repair antigen for 20 min. Then endogenous peroxidases were wiped off. Thereafter, tissues were incubated with 5% BSA for 30 min. Anti-PDGFRA (rabbit, 1:100, HuaBio, ET1702-49) was added in sections at 4 °C overnight. After washed with PBS for three times and probed with HRP conjugated compact polymer system for 30 min, DAB was used as the chromogenic agent. Hematoxylin was used to dye the cell nucleus.

### Statistical analysis and visualization

All statistical analysis were based on R (v4.0.5), and data visualization was performed on R packages Seurat (v4.1.0), ggplot2 (v3.3.5), ggsignif (v0.6.1), pheatmap (v1.0.12) and ggstatsplot (v0.9.1) [17].

## Results

### Spatial transcriptomics to identify intra-tumor heterogeneity in CRC

**Fig. 1:**
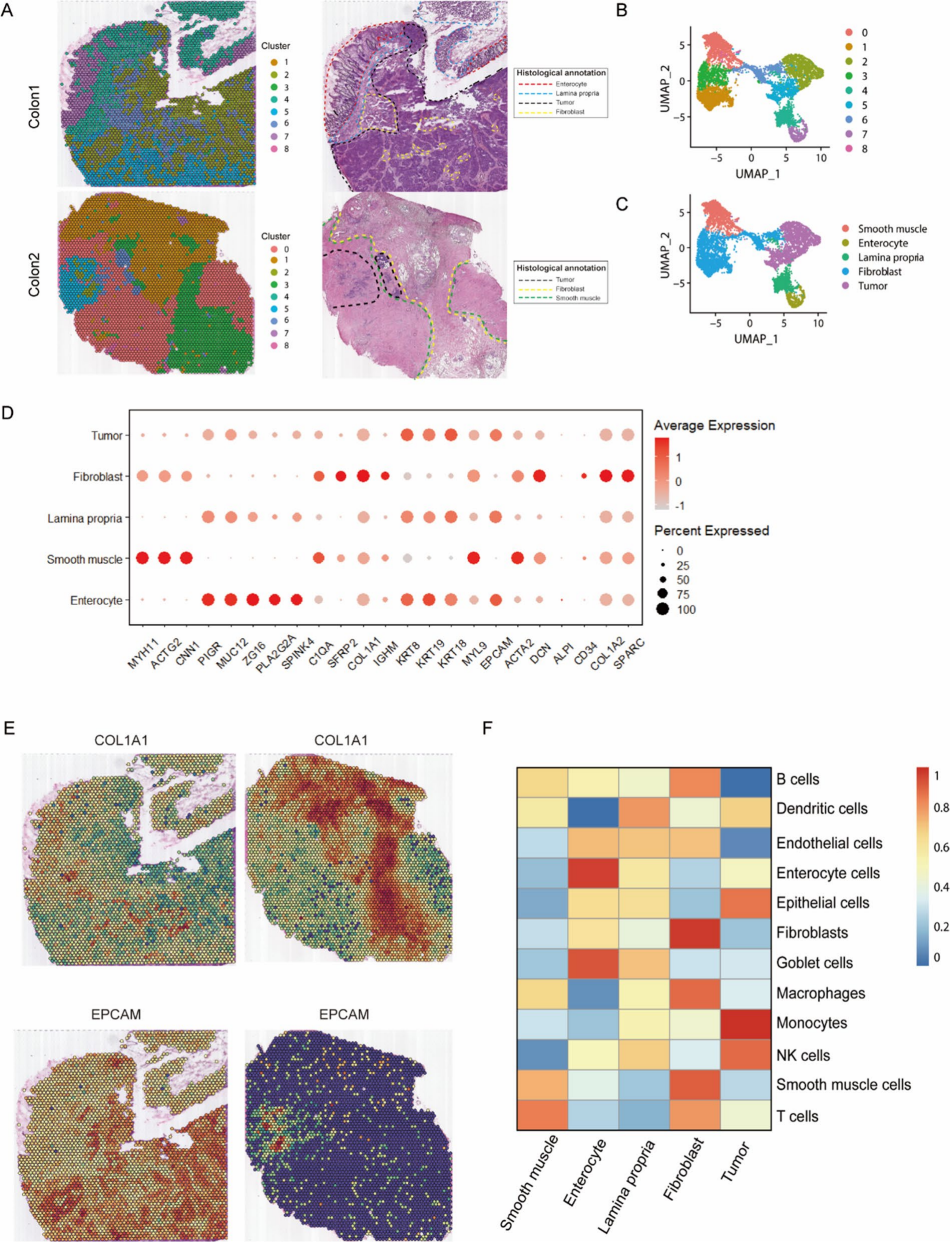
Spatial transcriptomics (ST) to identify intra-tumor heterogeneity in colorectal cancer. **A** Spatial images of unsupervised clustering results (left) and hematoxylin–eosin staining (H&E) sections annotations (right). **B**, **C** UMAP plot of cell types and PCA clustering result profiled in the presenting work. **D** The expression level of cell markers across different morphological regions through the Dot plot. **E** Spatial plots show the spatial expression pattern of fibroblast and tumor markers (COL1A1, EPCAM) in this study. **F** Heatmap of cell-type scores estimated by ssGSEA

In this study, we focused on two patients’ spatial transcriptomics data from a previously published CRC research (ST-P1, untreated, ST-P3, NACT with PR) [9]. Firstly, we used unsupervised clustering to cluster similar ST spots and divide into nine distinct clusters (Fig. 1A, B). Secondly, we annotated the clusters according to H&E sections and cell markers, then five morphological regions were identified (Fig. 1A, C, D). The results showed that unsupervised clustering analysis could effectively cluster ST spots with similar features into the same cluster, such as fibroblast and tumor regions (Fig. 1E). In addition, unsupervised clustering analysis could also subdivide tissues sections, which facilitates the discovery of tissue heterogeneity that is invisible to naked eye.

Because each spot of spatial transcriptomics contains more than one cell, its accuracy is lower than that of single-cell sequencing. Therefore, we scored well-defined genesets of 12 cell types [7, 18–24] by ssGSEA algorithm to recognize cell types that contained in each cluster. The annotation results had demonstrated the effectiveness of using ssGSEA in identifying cell populations of each cluster. As shown in Fig. 1F, fibroblast, enterocyte and smooth muscle were precisely annotated, and the lamina propria is composed of loose connective tissue that contains a variety of cells such as: dendritic cells, NK cells, etc. [25], while the tumor region was significantly enriched with monocytes, NK cells, and epithelial cells, indicating the existence of immune-inflammatory microenvironment in tumor region, which was consistent with previous studies [4, 5]. In all, spatial transcriptome analysis combined with ssGSEA can accurately determine the cell types contained in cell populations and offset the insufficiency of resolution in ST.

### CAFs-enriched subgroups are identified in CRC

**Fig. 2:**
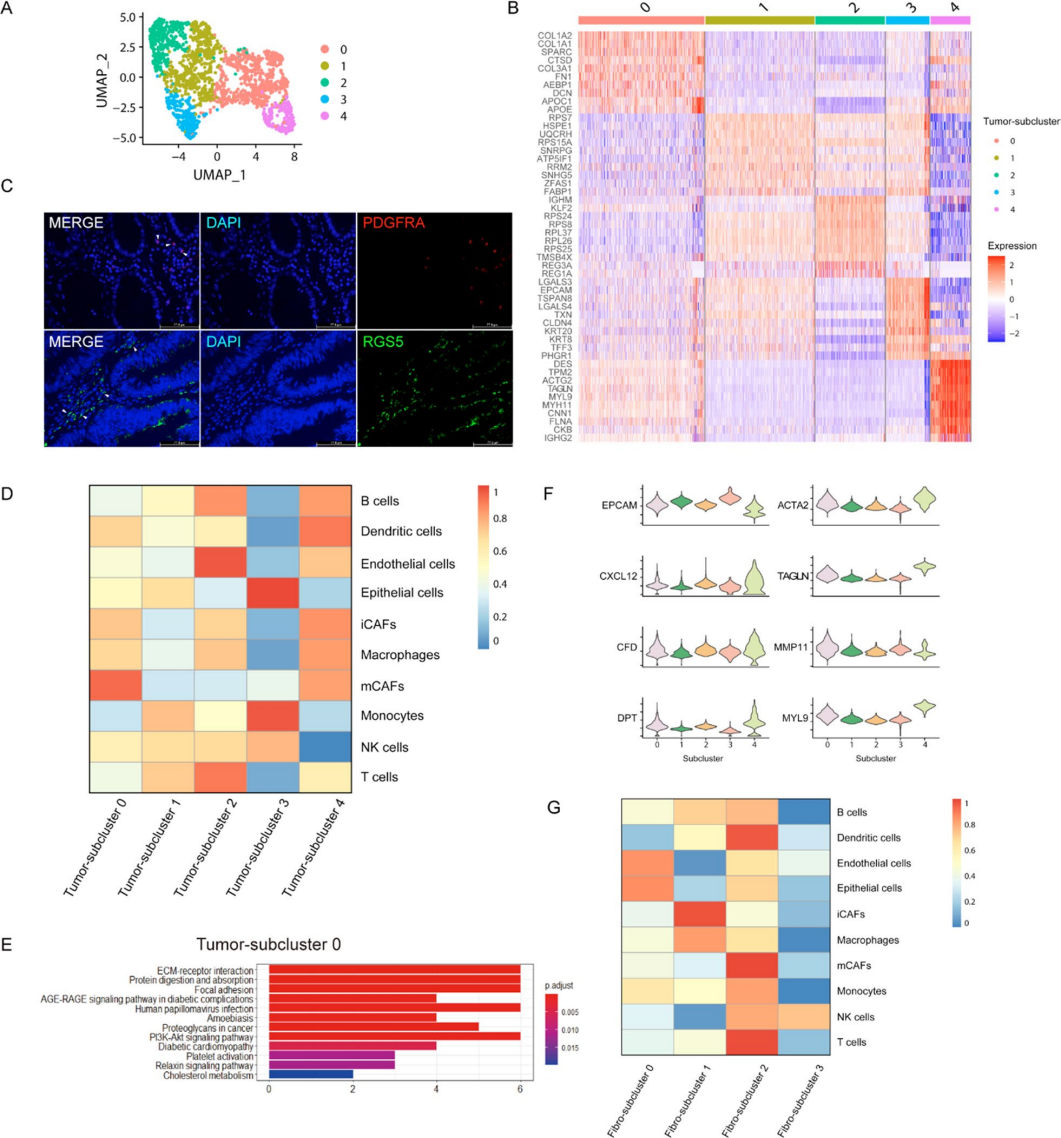
CAFs-enriched subgroups are identified in CRC. **A** UMAP plot of unsupervised clustering result of tumor subclusters. **B** Heatmap of differentially expressed genes (DEGs) of different Tumor-subclusters. **C** Immunofluorescence analysis of specific proteins expression of iCAFs and mCAFs. **D** Heatmap of tumor microenvironment (TME) cell-type scores calculated by ssGSEA in tumor region. **E** KEGG enrichment functions of up-regulated genes in Tumor-subcluster 0. **F** Violin plots show the expression of tumor, inflammatory-cancer-associated fibroblasts (iCAFs) and myo-cancer-associated fibroblasts (mCAFs) markers in tumor region. **G** Heatmap of tumor microenvironment (TME) cell-type scores calculated by ssGSEA in fibroblast region

To analyze the heterogeneity of tumor region, principal component analysis (PCA) was used to cluster tumor region into 5 subclusters (Tumor-subcluster 0 ~ 4) (Fig. 2A). To find the cell types and functions of each cluster, we analyzed cluster-specific differential expression genes (DEGs) and found that subcluster 0 highly expressed fibroblast markers, such as: COL1A2, COL1A1, SPARC and COL3A1, etc. (Fig. 2B). Therefore, we considered subcluster 0 as CAFs-enriched cluster. Li et al. [19] previously identified two types of CAFs at single-cell sequencing of CRC, in which CAF-A expressed genes related to ECM remodeling, while CAF-B expressed cell markers of myo-fibroblasts, such as ACTA2 and TAGLN. Elyada et al. [6] identified two distinct types of CAFs from human pancreatic cancer, named mCAFs and iCAFs, an emerging study [7] also confirmed the presence of two different fibroblasts subtypes in bladder cancer. As seen in Fig. 2C, to confirm the existence of CAFs with specific signatures, we applied immunofluorescence analysis and we detected the expression of PDGFRA (iCAFs marker) and RGS5 (mCAFs marker) in colorectal cancer [7]. Thereafter, we utilized ssGSEA to further investigate the cell subtype of subcluster 0. And the result showed that subcluster 0 enriched with mCAFs (mCAFs-enriched cluster) (Fig. 2D, F).

To determine the functions of mCAFs-enriched cluster, we carried out KEGG pathway enrichment analysis. As shown in Fig. 2E, mCAFs-enriched cluster (Tumor-subcluster 0) was related to ECM-receptor interaction, focal adhesion, and proteoglycans in cancer et al., suggesting that mCAFs has the function of ECM remodeling in TME, which was consistent with previous studies [6, 7, 26]. It was proved again that ssGSEA was robust for spatial transcriptomics data.

**Fig. 3:**
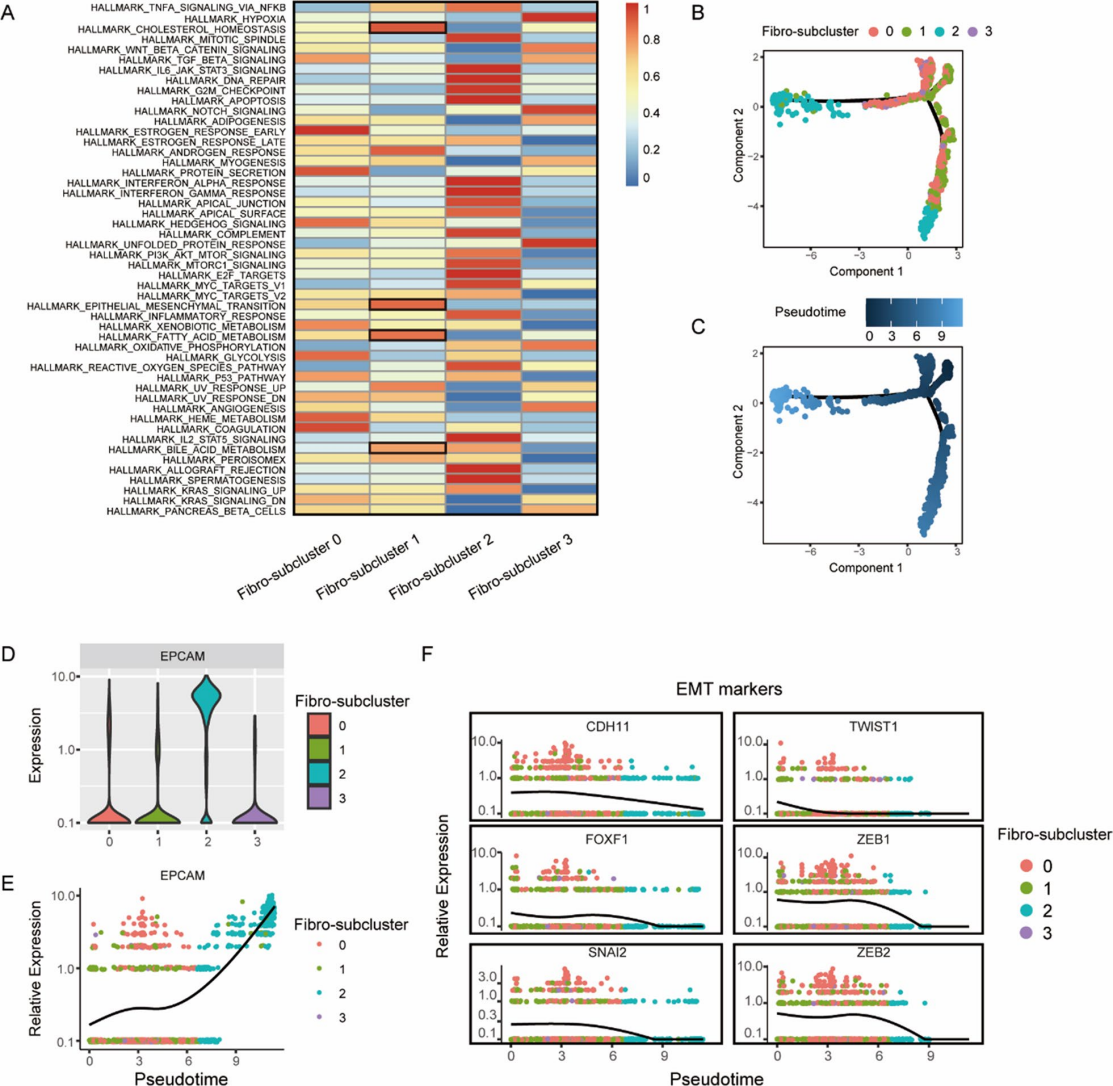
iCAFs promote oncogenesis by altering tumor microenvironment. **A** Heatmap shows ssGSEA result to explore the functional roles of different fibro-subclusters. **B**, **C** Trajectory of differentiation of different Fibro-subclusters predicted by monocle 2. **D**, **E** The differential expression of EPCAM in distinct Fibro-subclusters. **F** Gene expression level in single spot ordered along the pseudotime for EMT markers

In addition, we also conducted in-depth analysis on fibroblast region (Additional file 1: Fig. S1). The result showed that Fibro-subcluster 1 enriched iCAFs, named iCAFs-enriched cluster, and Fibro-subcluster 2 enriched mCAFs (Fig. 2G), because it highly expressed EPCAM, thus we identified it as mCAFs-enriched tumor cluster (Fig. 3D, E). Interestingly, when subclusters annotated by cell markers, we found that marker of CAFs, such as RGS5 and ACTA2 in mCAFs, PDGFRA and CXCL12 in iCAFs, were not specifically expressed in a certain subcluster (Fig. 2F, Additional file 1: Fig. S1D). Therefore, it further indicates that the accuracy of 10× spatial transcriptome data is limited, and spatial transcriptomics combined with multidimensional analysis (such as ssGSEA) can provide more detailed information. In detail, we found that anti-tumor immune cells such as T cells and dendritic cells were significantly enriched in mCAFs-enriched tumor cluster, iCAFs and macrophages were co-enriched in iCAFs-enriched cluster, however the anti-tumor immune cells, especially NK cells, reduced significantly in iCAFs-enriched cluster (Fig. 2G). It has been reported that CAFs can inhibit the immune system by releasing cytokines, chemokines and other compounds, thus leading to tumor metastasis [7, 27], indicating the important role of iCAFs in the immune microenvironment and its value as an anti-cancer drug target.

### iCAFs promote oncogenesis by altering tumor microenvironment

We applied ssGSEA enrichment analysis on fibroblast region to reveal the functions of iCAFs in TME, and our results indicated that iCAFs were associated with epithelial mesenchymal transition (EMT), cholesterol homeostasis, bile acid metabolism, and fatty acid metabolism (Fig. 3A). To further explore the transitional relationships of EMT phenotype in distinct clusters (Fig. 3B, C), we examined the expression level of EMT markers in different clusters through pseudotime analysis (Fig. 3F). Strikingly, the results demonstrated an increased expression of EMT signatures in iCAFs-enrich cluster, while lack of expression in tumor site (Fibro-subcluster 2). These results suggest that CAFs, rather than tumor epithelial cells, might be the main culprit in promoting EMT and leading to tumor metastasis [19]. Metabolic reprogramming also plays an important role in tumor proliferation and metastasis [28]. In this study, iCAFs were supposed to be correlated with lipid metabolism through the bioinformatics analysis (Fig. 3A), suggesting that the changes of lipid metabolic activity of iCAFs in TME might be a potential mechanism in promoting tumor tumorigenesis.

### Chemotherapy alters the tumor microenvironment

**Fig. 4:**
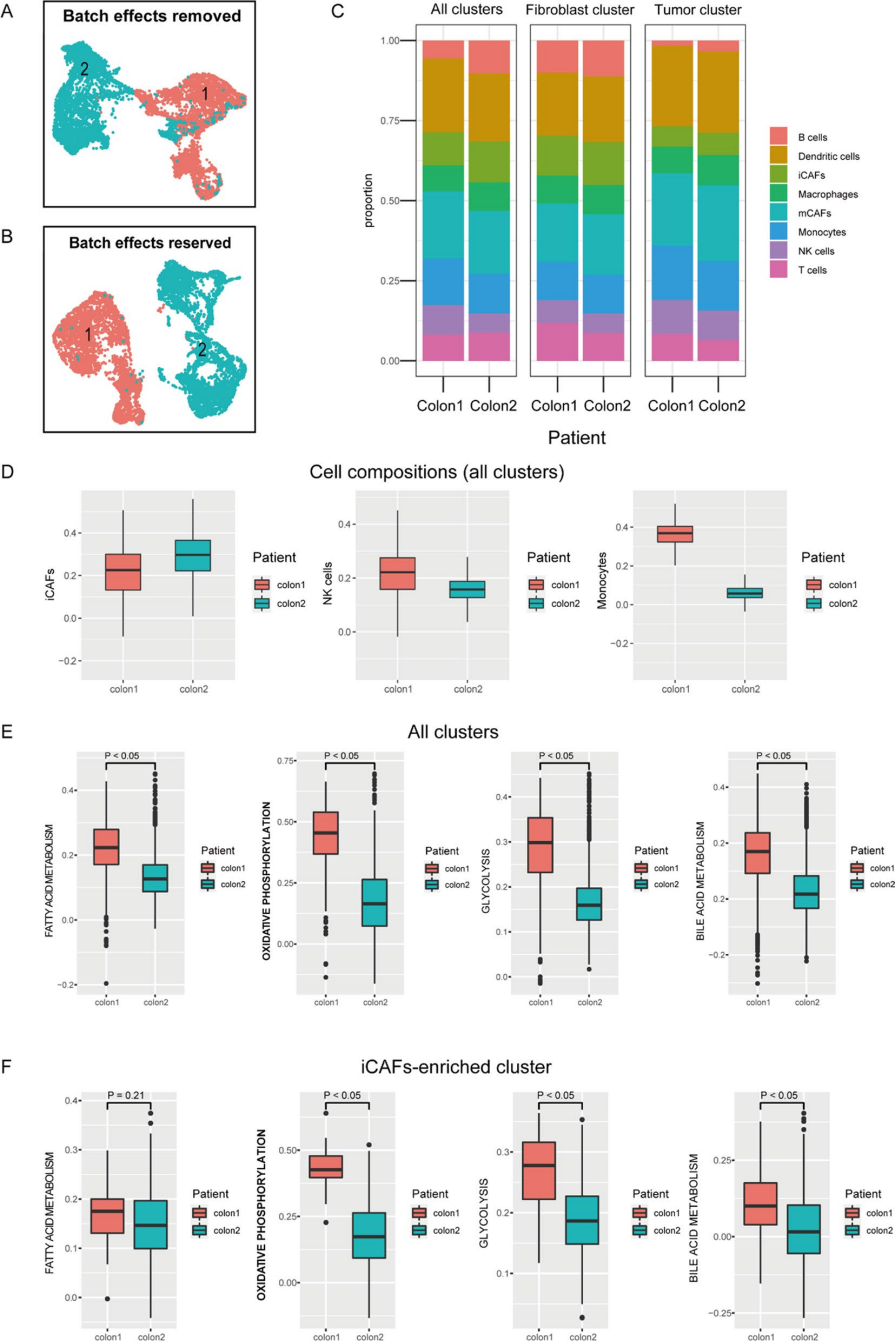
Chemotherapy alters the tumor microenvironment. **A**, **B** UMAP plots show patient-specific spatial transcriptomics (ST) expression patterns between colon1 and colon2, **A** UMAP plot of ST data after CCA, **B** UMAP plot of ST data without CCA. **C** Cell compositions predicted in different patients by ssGSEA, predicted cell compositions in all clusters (left), predicted cell composition in fibroblast cluster (middle), predicted cell composition in tumor cluster (right). **D** Boxplots show the cell compositions of iCAFs, NK cells and monocytes in all clusters. **E**, **F** Box plots show the patient-specific metabolism patterns scored by ssGSEA.* p* value < 0.05 was considered as cut-off criteria

When we integrated two patients’ datasets, we found a patient-specific ST expression pattern (Fig. 4B), so we used CCA [10] to remove the batch effects on ST data. It is noteworthy that there was still obvious heterogeneity between colon1 and colon2 (Fig. 4A), therefore we supposed that chemotherapeutic drugs may be one reason for explaining the differential expression profiling in TME. In detail, we analyzed the cell compositions of two patients, results showed that the putative proportion of iCAFs relatively increased in all clusters (Fig. 4C left, Fig. 4D and Additional file 1: Figs. S2, S3) of colon2 (NACT with PR), while the proportions of some anti-tumor immune cells decreased, such as: NK cells, monocytes, etc. (Fig. 4C left, Fig. 4D and Additional file 1: Figs. S2, S3). In addition, we also examined the metabolism pattern changes caused by chemotherapeutic drugs, and we found that the metabolic activity significantly decreased in colon2 (Fig. 4E). However, when we focused on iCAFs-enriched cluster, we detected that fatty acid metabolic activity did not decrease in colon2 (Fig. 4F), then we supposed this phenomenon may be associated with iCAFs. Perhaps, it is the decrease of these tumor-killing cells and metabolism patterns changes caused by iCAFs that explain the internal mechanisms of drug resistance.

### iCAFs is associated with clinical prognosis and immune infiltration

**Fig. 5:**
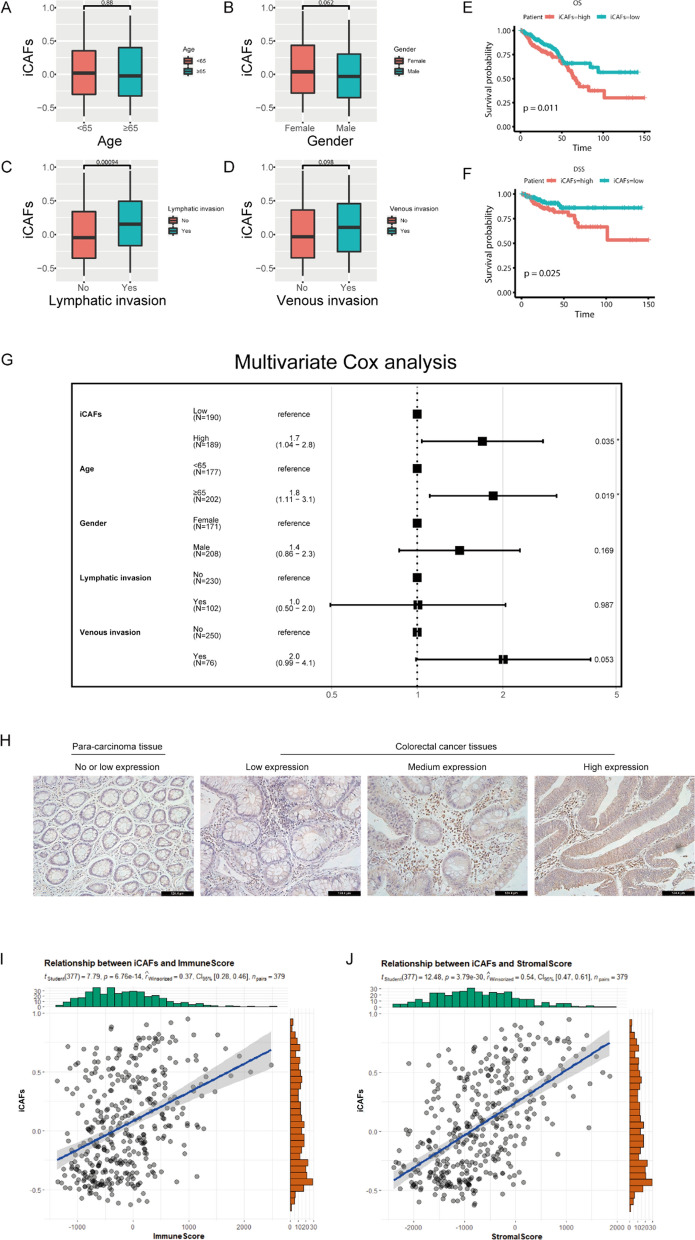
iCAFs is associated with clinical prognosis and immune infiltration. **A**–**D** Box plots show the correlations between iCAFs and clinicopathologic features. **E**, **F** K-M survival plots show that high iCAFs predicted poor prognosis in TCGA COADREAD cohort, OS: overall survival, DSS: disease specific survival. **G** Forest map shows that iCAFs is an independent prognostic factor for OS. **H** Immunohistochemical analysis of PDGFRA expression in colorectal cancer or para-carcinoma tissues. **I**, **J** The diagrams show the relationships between iCAFs and immune score as well as stromal score. *p* value < 0.05 was considered as cut-off criteria

To correlate spatial transcriptomics data with public dataset, we evaluated the clinical significance of iCAFs in TCGA COADREAD cohort. Clinicopathological analysis showed that iCAFs were significantly associated with lymph node invasion (*p* < 0.05), the higher of iCAFs, the more possibility of lymph node invasion (Fig. 5A–D). In addition, univariate (Fig. 5E, F) and multivariate Cox proportional hazards regression (Fig. 5G) were used to analyze the association of the accumulation of iCAFs with prognosis. Multivariate analysis after adjusting confounding factors showed that the accumulation of iCAFs was an independent prognostic factor for OS (*p* = 0.035) (Fig. 5G). To explore the differential expression of iCAFs in distinct tissues, we specially detected the expression of iCAFs marker (PDGFRA) in tumor tissues and para-carcinoma tissues (n = 5) using immunohistochemical method, and our results demonstrated that the higher proportion of iCAFs in tumor tissues rather than para-carcinoma tissues (Fig. 5H). These results further indicated that iCAFs were associated with poor prognosis. When we focused on the relationship between iCAFs and immune inflammation, ESTIMATE algorithm was applied to calculate the immune score and stromal score for TCGA cohort, and the results showed that iCAFs was significantly correlated with immune score (r = 0.39, *p* < 0.05) (Fig. 5I, J), suggesting that iCAFs could interact with immunosuppressive cells [27, 29], thus inhibit the anti-tumor inflammatory response in TME.

**Fig. 6:**
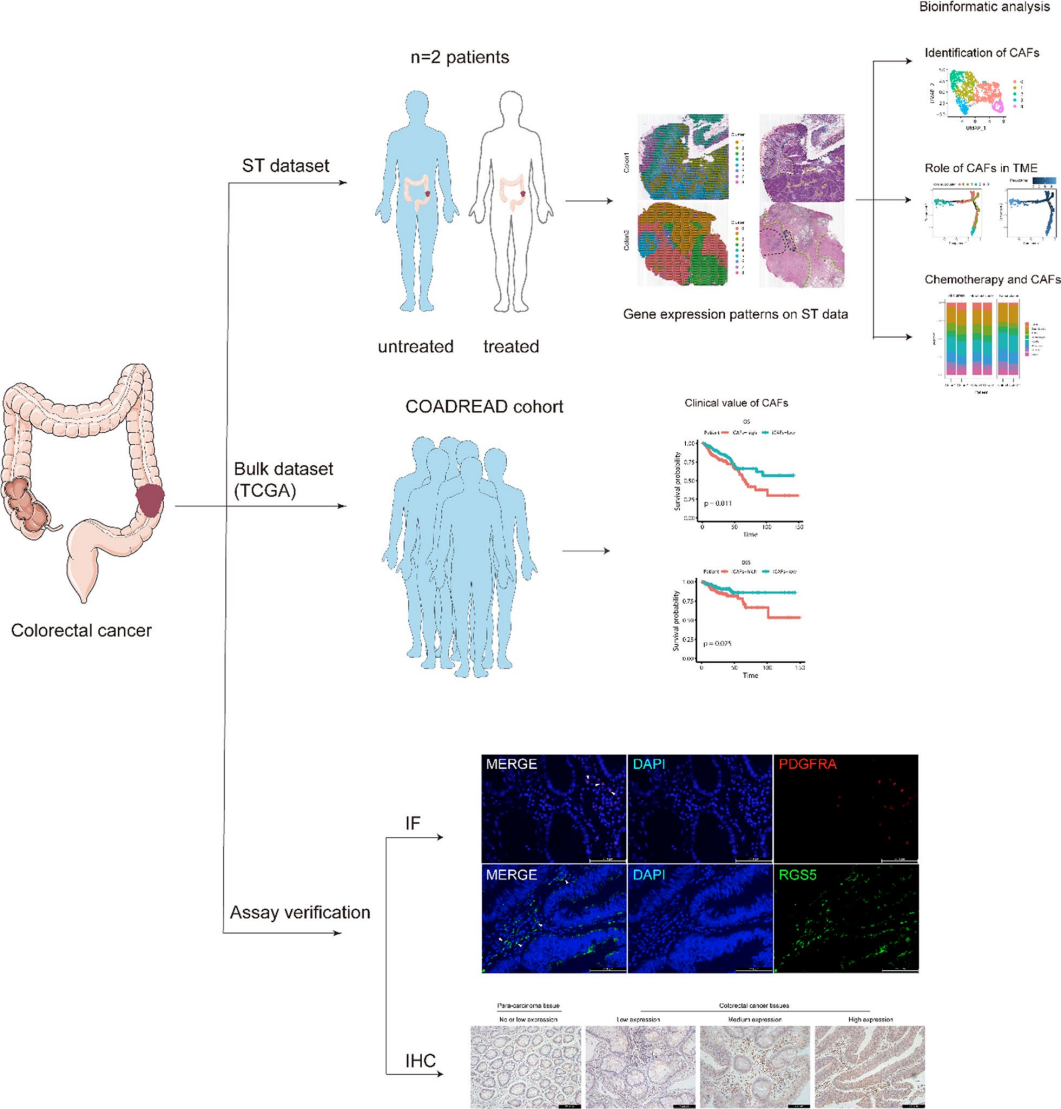
Workflow of bioinformatic analysis. Two types of datasets were utilized in this project. ST data was used to explore the role of CAFs in TME within patients with colorectal cancer, while bulk RNA-seq dataset was applied to validate the clinical translational value of CAFs identified in ST data. Then immunofluorescence staining and immunohistochemical was applied to validate the results based on bioinformatics analysis and we confirmed the functions of two CAFs types in CRC

## Discussion

Currently, targeted drugs combined with surgery has largely reduced the mortality of colorectal cancer, however tumor metastasis and drug resistance are still important factors leading to poor prognosis of CRC patients [8], so new therapeutic strategies are waiting to be discovered. Previous studies pay more attention to the biology of tumor cells, nevertheless, emerging research gradually noticed the important roles of tumor microenvironment in tumorigenesis, metastasis and drug resistance [5, 30], while CAFs as the main stromal components in the microenvironment, can affect the tumor growth and metastasis through various mechanisms [3]. In this research, we utilized spatial transcriptome data and public TCGA cohort dataset to elucidate CAFs functions and its interactions with the microenvironment, hoping to promote the development of drug treatment strategies, a workflow was drew to clearly show the overview of our project (Fig. 6).

Li et al. firstly divided CAFs into two distinct subpopulations (CAF-A and CAF-B) based on the single-cell sequencing data in CRC. CAF-A was related to ECM remodeling, while CAF-B was similar to myo-fibroblasts. However, the functional roles of these two types of CAFs remain unclear [19]. In recent years, studies have classified CAFs into iCAFs and mCAFs according to their functions in pancreatic cancer, prostate cancer and triple negative breast cancer [6, 7, 31]. We also identified the existence of these two types of CAFs in CRC with fundamental experiment. An increasing number of research reveal that CAFs through various approaches such as receptor activation, cytokine and cytotoxic production, to inhibit NK cells [3, 27, 32], and we also found that NK cells were decreased in iCAFs-enriched cluster, which was consistent with these studies. In addition, we detected that iCAFs and macrophages were co-enriched in the same subcluster by using bioinformatics analysis. Zhang et al. reported that CAFs can inhibit NK cells proliferation by regulating tumor-associated macrophages (TAM) [29], suggesting the extensively interactions between iCAFs and immune cells in TME. Besides, the relationships of chemotherapy drugs and tumor microenvironment were also examined, and we found that the proportion of iCAFs increased while the number of NK cells and monocytes decreased in patient who underwent neoadjuvant chemotherapy (colon2). Since iCAFs can inhibit immune cells in TME, the decrease of immune cells may be related to iCAFs [27]. Therefore, we speculated that the increase of iCAFs in patient who underwent chemotherapy might be an important mechanism for the occurrence of drug resistance. In the future, more fundamental experiments should be conducted to elucidate the underlying mechanism.

Although epithelial-mesenchymal transformation (EMT) has been widely studied, we still have limited understanding of the mechanism of this phenomenon [33]. Our study indicated that iCAFs up-regulated EMT signature, while tumor cells lacked of an EMT marker, suggesting that EMT may be driven by fibroblasts rather than tumor cells [19, 33, 34]. In addition, metabolic reprogramming is also an important mechanism for tumor progression. More and more studies have shown that CAFs can promote tumor metastasis through the metabolic crosstalk with tumor cells or other stromal cells in TME [28, 35]. Despite the known glycolysis and amino acid metabolism play important roles in TME, there are emerging evidences demonstrate that lipid metabolism is also essential for tumor development [36, 37]. This is consistent with our ssGSEA pathway analysis. Furthermore, when we investigated the relationship between chemotherapy and metabolism, we discovered opposite trends between fatty acid metabolism pattern and other metabolism patterns in iCAFs-enriched cluster, and we speculated this phenomenon might lead to drug resistance. Therefore, targeted therapy toward iCAFs as well as its metabolites in tumor microenvironment is likely to be an effective way to inhibit tumor metastasis.

By correlating our spatial transcriptomics results to a public database, we confirmed that iCAFs was associated with immune infiltration and clinical outcomes in CRC. As main stromal cells in tumor microenvironment, CAFs can be detected in almost all solid tumors, thus targeting iCAFs can include the vast majority of patients, which is an ideal choice for colorectal cancer treatment.

There are still some limitations in this project. In this study, spatial transcriptome data were used to profile the heterogeneity of TME. Although this emerging technology can provide spatial location information of cells and facilitate the identification of cell types, due to the fact that 10X Genomics platform contains 1–10 cells in each spatial spot [38], the accuracy of spatial transcriptome data is lower than that of single-cell sequencing, so more efforts needed to improve the resolution of ST. Therefore, aiming to annotate cell types of each cluster more precisely, ssGSEA algorithm were applied in this project. Compared with morphological regions annotation, each cluster had been accurately annotated, which once again confirmed that ssGSEA algorithm is feasible for ST. Hence, the combination of ST and ssGSEA can provide a more comprehensive understanding of the cell types contained in the subpopulation, so as to discover the co-enriched cell types and the potential interactions between themselves. In addition, the small sample in our study may also be a possible limitation. And our project concerned on the functions of iCAFs in TME, the value of mCAFs was not fully recognized, so ongoing efforts are required to explore the functions of mCAFs.

## Conclusions

In summary, our studies identified two different types of CAFs in CRC and provided an in-depth perspective on the interactions between CAFs and the tumor microenvironment. This study provides a practicable idea for drug therapy strategies targeting CRC in the future.

## Supplementary Information

**Additional file 1.** Figures S1–S3, Tables S1–S9.

## Data Availability

Colorectal cancer spatial transcriptomics dataset analyzed in this research can be found at: http://www.cancerdiversity.asia/scCRLM/. Bulk transcriptomics of colorectal cancer comes from TCGA cohort COADREAD at: https://xena.ucsc.edu/.

## Abbreviations

ST: Spatial transcriptomics
CAFs: Cancer-associated fibroblasts
TME: Tumor microenvironment
mCAFs: Myo-cancer-associated fibroblasts
iCAFs: Inflammatory-cancer-associated fibroblasts
CRC: Colorectal cancer
ssGSEA: Single sample gene set enrichment analysis
ECM: Extracellular matrix
NACT: Neoadjuvant chemotherapy treatment
TCGA: The Cancer Genome Atlas
DEGs: Differentially expressed genes
GSVA: Gene Set Variation Analysis
PCA: Principal component analysis
EMT: Epithelial mesenchymal transition
PR: Partial response
TAM: Tumor-associated macrophages
H&E: Hematoxylin–eosin staining
IF: Immunofluorescence
IHC: Immunohistochemistry
